# The Feasibility of the Repeated Administration of Acetylsalicylic Acid Mini-Tablets to Children with Kawasaki Disease: A Pilot Study

**DOI:** 10.3390/pharmaceutics17030333

**Published:** 2025-03-05

**Authors:** Noriko Hida, Fuka Serizawa, Takehiko Sambe, Akihiro Nakamura, Tsutomu Harada

**Affiliations:** 1 Department of Clinical Research and Development, Graduate School of Pharmacy, Showa University, Tokyo 157-8577, Japan; 2 Department of Pharmaceutics, Graduate School of Pharmacy, Showa University, Tokyo 157-8577, Japan; gp22-f011@pharm.showa-u.ac.jp (F.S.); hironak@pharm.showa-u.ac.jp (A.N.); tharada@pharm.showa-u.ac.jp (T.H.); 3 Research Administration Center, Showa University, Tokyo 142-8555, Japan; t-sambe@med.showa-u.ac.jp

**Keywords:** acetylsalicylic acid, mini-tablet, Kawasaki disease, pediatric formulation, in-hospital preparation, acceptability, adherence

## Abstract

**Background/Objectives**: Mini-tablets are a novel pediatric dosage form designed to mask drug taste and facilitate easier administration. This study aimed to assess the feasibility and acceptability of uncoated acetylsalicylic acid (ASA) mini-tablets in Japanese children with Kawasaki disease (KD) aged 1 to 4 years. **Methods**: A retrospective case series study of three pediatric patients with KD treated with ASA mini-tablets (3 mm diameter, 10 mg) was conducted at Showa University Hospital. ASA mini-tablets were administered for up to 68 days. Caregivers recorded daily medication intake and any issues in medication logbooks. **Results**: All three patients successfully took 100% of the prescribed doses. No adverse events related to mini-tablet ingestion were reported. Patients could take the mini-tablets for extended periods (63–68 days) as part of their KD treatment. **Conclusions**: ASA mini-tablets showed potential acceptability in this small cohort of pediatric patients with KD. This study represents the first investigation into the acceptability of mini-tablets containing active ingredients in Japanese pediatric patients. Larger studies are needed to confirm these findings and evaluate long-term safety and efficacy.

## 1. Introduction

Ensuring access to appropriate pediatric medicine led the World Health Organization in 2008 to propose solid oral dosage forms as the preferred formulation for pediatric use [1]. In Japan, the most commonly used pediatric dosage forms are syrup, fine granules, dry syrup, and orally disintegrating tablets [2,3]. Saito et al. [3] reported that for patients younger than 10 years (*n* = 354), powders such as fine granules, granules, and dry syrups are most frequently prescribed (*n* = 252, 71.2%). For children under 6 years, although powders remain the most common form of prescription, liquids such as syrups and suspensions are the second most frequently prescribed dosage form (*n* = 60, 21.0%). The frequency of tablet prescriptions increases as children grow, but remains relatively uncommon. In Japan, pharmacists typically dispense a single dose of powder into a sachet. The powder is then consumed by the patient in an unaltered state; alternatively, the parent or caregiver may add a small quantity of water or syrup to create a paste or kneaded syrup for the child to ingest [4]. However, children may refuse to take medication in powder or paste form due to its taste and texture [5], creating challenges for both healthcare providers and caregivers.

Mini-tablets represent a novel pediatric dosage form under development in Europe and the United States. These mini-tablets have the potential to prevent immediate disintegration in the oral cavity and effectively mask the taste of the drug [6,7]. Children under the age of 5 generally lack the ability to safely swallow solid capsules or tablets larger than 10 mm [8]. Moreover, global research on mini-tablets has advanced, and studies in Japan have shown that 2 mm mini-tablets are well accepted by children over 6 months old [9,10]. Similarly, in other countries, mini-tablets are found to be more acceptable for children aged 6 months to 6 years [11,12]. Children aged 2–7 years can ingest film-coated mini-tablets with soft food or water without adverse effects or dysphagia [13]. Additionally, rectangular tablets have been suggested as a safe alternative to liquid formulations and multiple mini-tablets for children aged 1–5 years [14]. Mini-tablets are increasingly seen as a practical option for pediatric patients due to their ability to provide precise dosing and safe ingredients [15]. However, previous studies have primarily investigated placebos, and it remains unclear whether mini-tablets containing active ingredients are equally well tolerated. Recent studies have demonstrated the acceptability of 3 mm mini-tablets in pediatric populations [9,12,13,14]. For example, Klingmann et al. [12] reported a 99% success rate in swallowing 3 mm mini-tablets among children aged 2–7 years, with no adverse events. Münch et al. [14] found that children aged 2–7 years could ingest 3 mm film-coated mini-tablets with soft food or water without experiencing adverse effects or dysphagia. Similarly, Thomson et al. [16] showed that children aged 2–6 years could successfully swallow a single 3 mm mini-tablet, with acceptability increasing with age. However, these studies focused primarily on placebos.

ASA is a white crystalline substance, often formulated in granular or powder form, with a slightly acidic and sour taste when taken orally [17]. Acetylsalicylic acid (ASA) gradually hydrolyzes into salicylic acid and acetic acid, contributing to its strong, distinctive taste. This degradation can occur during storage at medical institutions or in homes. In Japan, it is not uncommon for children with Kawasaki disease (KD) to be prescribed ASA for extended periods [18]. ASA is expected to demonstrate anti-inflammatory effects in the acute phase and inhibit platelet aggregation, making it a cornerstone of treatment. Rapid suppression of the intense inflammatory response during the acute phase is crucial to prevent the formation of coronary aneurysms [19].

KD is an acute, self-limited vasculitis that primarily affects children under 5 years of age. It presents with symptoms such as fever, rash, conjunctival injection, changes in the extremities, oral mucosal changes, and cervical lymphadenopathy. KD is the leading cause of acquired heart disease in children in developed countries, with coronary artery aneurysms being the most serious complication [20,21,22,23]. In Japan, KD is particularly prevalent, with an annual incidence of approximately 330 per 100,000 children under five [20]. The etiology of KD remains unknown, but it is thought to involve a complex interplay between genetic factors and environmental triggers. Early diagnosis and prompt treatment with intravenous immunoglobulin and ASA are crucial for reducing the risk of coronary artery complications [18,19].

We previously created mini-tablets capable of masking the taste and odor of ASA and conducted a pharmacokinetic study in healthy adults. This study confirmed that the mini-tablets demonstrated comparable dissolution properties and bioequivalence to ASA powder and mini-tablets [24]. However, data on the long-term administration of ASA mini-tablets in Japanese pediatric patients are lacking. This case series aimed to evaluate the clinical feasibility of the repeated administration of ASA mini-tablets in children with KD. Rather than conducting a full clinical trial, this pilot study serves as an initial evaluation of the feasibility of repeated ASA mini-tablet administration in a pediatric population. This study presents an initial evaluation of the acceptability of mini-tablets containing active ingredients in Japanese pediatric patients, addressing the unique challenges of medication administration to children with KD.

## 2. Materials and Methods

### 2.1. Material

ASA mini-tablets (3 mm diameter, 10 mg) were prepared at the Pharmacy Department of Showa University Hospital according to the method described by Hida et al. [24]. The formulation consisted of the following:

- Active pharmaceutical ingredient (API): Acetylsalicylic acid (ASA) 10 mg (Japanese Pharmacopoeia grade, Yoshida Pharmaceutical Co., Ltd., Tokyo, Japan).
- Excipients: Crystalline cellulose (Japanese Pharmacopoeia) (2 mg), corn starch (Japanese Pharmacopoeia) (2 mg), D-Mannitol (Japanese Pharmacopoeia) (5.4 mg), magnesium stearate (Japanese Pharmacopoeia) (0.6 mg).

This formulation was designed considering the balance of formulation characteristics and ease of manufacturing, ensuring it was suitable for administration to pediatric patients. Acetylsalicylic acid, as an active ingredient, exhibited anti-inflammatory and antiplatelet effects. Crystalline cellulose (CEOLUS™ KG1000, Asahi Kasei Corporation, Tokyo, Japan) was used as a binder and diluent, providing good compressibility and flowability. Corn starch (Yoshida Pharmaceutical) was used as a disintegrant to promote the rapid disintegration of the tablet. D-Mannitol (Parteck^®^ M200, Merck KGaA, Darmstadt, Germany) was used as a diluent, providing sweetness and increasing tablet hardness. Magnesium stearate (Taihei Chemical Industrial Co., Ltd., Osaka, Japan) was used as a lubricant to prevent powder adhesion during tableting.

The mini-tablets were uncoated with a smooth surface and designed for pediatric use.

### 2.2. ASA Mini-Tablet Formulation and Preparation

#### 2.2.1. Manufacturing Process

The manufacturing process for ASA mini-tablets is outlined below:

Initially, 50 g of acetylsalicylic acid powder was measured and transferred into polyethylene bag #1. Subsequently, 10 g of cellulose crystals, 10 g of corn starch, and 27 g of mannitol were placed in a separate polyethylene bag, #2, and added to polyethylene bag #1 containing the acetylsalicylic acid. The mouth of polyethylene bag #1 was closed and, after sufficient air was added, the bag was rotated by hand 200 times to thoroughly mix the powder. After adding magnesium stearate (3 g), the polyethylene bag was closed, sufficient air was introduced, and the powder was mixed by rotating the bag 200 times. The mixed powder was then subjected to a visual inspection to ascertain the presence of any foreign matter. If any foreign matter was detected, it was removed, and the process was restarted with a new batch. The mixture was fed into a single-stroke tableting machine (N-30E, Okada Seiko Co., Ltd., Osaka, Japan) and compressed using 3 mm punches. The compression pressure was adjusted within the range of 750–1000 N to achieve a tablet weight of 20 mg. The target tablet hardness was set in the range of 1–2 kgf, with a goal of achieving over 1.5 kgf. The tableting speed was set at approximately 30 tablets per minute. After compression, the tablets were sieved to remove any adhering powder. Following quality control tests, approved tablets were placed in zip-lock aluminum bags with desiccants and stored. These tablets were stored in the investigational drug management room, where the temperature and humidity were kept constant.

The batch size was determined based on clinical trial requirements.

#### 2.2.2. Quality Control

A series of quality control tests were performed to ensure the quality of the ASA mini-tablets. The tests included an appearance inspection, a weight uniformity test, a tablet hardness test, a disintegration test, a dissolution test, and a content uniformity test. These tests were conducted in accordance with the Japanese Pharmacopoeia (JP).

The appearance inspection was conducted visually to check for any defects or inconsistencies. Weight uniformity was assessed by precisely measuring individual tablet weights. The mean weight of the mini-tablets was found to be 20.33 ± 0.57 mg, with a relative standard deviation (RSD) of 2.80%, indicating excellent consistency in drug content across the batch. Tablet hardness was evaluated by using a Kiyoshi digital hardness tester (KHT-40N, Fujiwara Seisakusho Co., Ltd., Osaka, Japan). The mean hardness of the six tablets measured was 1.63 kgf (standard deviation 0.16 kgf). The assay of uniformity of content was performed by taking three tablets as one increment and 10 random samples. Analysis by HPLC yielded an average content of 98.5% (standard deviation 2.76%) of the labeled amount (10 mg). The rationale behind selecting this method was that the recommended dosage is 3–5 mg/kg/day, and for a child weighing 10 kg, 3–5 tablets would be administered. The disintegration test was carried out using the JP method with purified water. The disintegration test involved the measurement of six tablets. The mean disintegration time was determined to be 130.0 s, with a standard deviation of 64.6 s. The dissolution test employed the paddle method with specified conditions, and content uniformity was determined using HPLC analysis.

To evaluate the stability of the ASA mini-tablets, an accelerated test was conducted at 40 °C and 75% relative humidity over a period of three months. The results showed an average drug residual rate of 98%, confirming the stability of the formulation under stressed conditions. To further ensure stability throughout the testing period, several measures were implemented, which included maintaining strict humidity control during the manufacturing process, packaging the mini-tablets in aluminum bags with desiccant, and providing instructions for use within a short period after opening. These precautions were taken to minimize exposure to environmental factors that could potentially affect the stability of the ASA mini-tablets.

### 2.3. Participants

This retrospective case series study included children diagnosed with KD at the Department of Pediatrics or Pediatric Cardiology at Showa University Hospital who were prescribed ASA mini-tablets after November 2022. Patient selection was based on the order of diagnosis, with the first three patients meeting the criteria being included in this pilot study. The patient selection period was from November 2022 to March 2023. Patients who were unable to maintain a medication logbook with the assistance of a parent or caregiver were excluded.

### 2.4. Eligibility

#### 2.4.1. Inclusion Criteria

- Patients diagnosed with KD according to the Japanese Circulation Society 2020 guidelines.
- Age range: 6 months to 6 years.
- Ability to swallow placebo mini-tablets during a screening test.

The age range for inclusion was set at 6 months to 6 years to include a wider variety of children. However, due to the specific clinical context and the availability of patients during the study period, the actual participants were limited to children aged 1 to 4 years.

#### 2.4.2. Exclusion Criteria

- Patients with dysphagia.
- Patients with a history or current peptic ulcer.
- Patients with a tendency to bleed.
- Patients with ASA asthma.
- Patients with ASA hypersensitivity.

### 2.5. Administration Method of Mini-Tablets

Mini-tablets can be administered either as a single dose or in divided doses, depending on the patient’s age and condition.

### 2.6. Survey Items

This study examined patients’ medical records and medication diaries for one year from the start of treatment. However, the present paper analyzes and reports only data from the initial treatment period (up to 69 days). This timeframe was deemed adequate for assessing the acceptability and safety of the mini-tablets as a pilot study, as it encompassed the acute treatment of KD and the initial recovery period.

Medical records and medication diaries were examined, with the following data collected: the clinical presentation of KD and administration of ASA mini-tablets at the outset of treatment and up to 1 year later.

#### 2.6.1. Information Collected from Medical Records

The dataset included information on sex, age at diagnosis, height, weight, and temperature; blood test results (serum sodium level, C-reactive protein level, white blood cell count, platelet count, brain natriuretic peptide level, aspartate aminotransferase level, and neutrophil count); the diameters of the right coronary artery, left main trunk, left anterior descending branch, and left circumflex branch by echocardiography; and information on the dosage and formulation of ASA prescriptions and the administration of intravenous immunoglobulin (IVIG) therapy. When IVIG therapy was administered, the recorded dosage was also included. In addition, a prediction score for IVIG treatment resistance was included in the dataset.

#### 2.6.2. Information Collected from Medication Logbooks

The dataset was compiled from information recorded in medication logbooks. The following data were collected:

(1) Date.
(2) Mood before and after ingestion of the mini-tablets (good, normal, or bad). In the event of non-compliance, what was the reason for this (e.g., chewed, swallowed, or refused)?
(3) Were the mini-tablets taken all at once, divided into multiple doses, or one tablet taken at a time?
(4) Description of the beverages consumed in conjunction with mini-tablets.

It is important to note that the medication intake data were collected through self-reporting by caregivers in medication logbooks. Therefore, the accuracy of the data relies on the caregivers’ recording practices and may be subject to recall bias.

### 2.7. Ethical Considerations

This study was conducted in accordance with the ethical principles set forth in the Declaration of Helsinki and ethical guidelines for human participants. The Showa University Research Ethics Review Board approved this study (approval number: 2024-051-A).

## 3. Results

### 3.1. Case Presentation

The medical records of the first three patients (two boys and one girl) diagnosed with KD and followed up at Showa University Hospital were reviewed. All patients were of Japanese nationality. The clinical characteristics of the patients at the time of referral to Showa University Hospital are presented in Table 1.

### 3.2. Treatment Outcomes and Safety

All three patients had high adherence rates (98.4–100%) to the ASA mini-tablet regimen (Table 1). Case 1 received mini-tablets during the maintenance phase, whereas Cases 2 and 3 started with the powder formulation. Dosing patterns varied, with Cases 1 and 2 predominantly taking single doses (52 and 11 days, respectively), while Case 3 consistently took divided doses. As in previous study, for patients aged 6–11 months, mini-tablets were primarily administered one at a time, while for patients aged 12–23 months, 4–5 mini-tablets were often administered simultaneously [9]. Patients used various beverages, including hojicha (roasted green tea), water, orange juice, and vegetable juice. No adverse events related to mini-tablet use were reported, and all patients showed no coronary artery dilation at the end of treatment.

### 3.3. Case 1

A 1 year and 11-month-old boy was referred to Showa University Hospital with fever and ocular conjunctival hyperemia. Additional symptoms included lip redness, cervical lymphadenopathy, and an indeterminate rash. Meeting five of the principal KD symptoms outlined in the KD Diagnostic Guide (sixth edition), the patient was diagnosed with KD and admitted to the hospital. On the day of admission, he was administered a loading dose of ASA (360 mg/day, prescribed as mini-tablets) and 25 g/day γ-globulin. He took 36 mini-tablets per day, divided into three doses of 12 mini-tablets every 8 h intervals. The fever subsided on the second day, leading to the discontinuation of γ-globulin treatment. By the fourth day, the fever had completely resolved, and the ASA dosage was reduced to a maintenance dose of 60 mg/day. The patient took six mini-tablets per day as a single dose for 52 days and in divided doses for 16 days. The patient was discharged from the hospital in good health and advised to continue ASA mini-tablets for 60 days post-discharge.

During treatment, transient first-degree and Wenckebach-type second-degree atrioventricular block were observed, but echocardiographic assessments remained normal. No electrocardiographic abnormalities or coronary artery dilation were detected during outpatient follow-up.

The patient missed the prescribed dose on the 32nd day but resumed medication the following day, completing the full prescription one day later than scheduled. He initially chewed the mini-tablets during the sixth dose but subsequently swallowed them without issues. No swallowing difficulties or safety concerns were reported.

### 3.4. Case 2 

A 4-year-old boy presented as an outpatient at Showa University Hospital with a fever. The patient displayed bilateral ocular conjunctival hyperemia, red lips, non-purulent lymphadenopathy, an indeterminate rash, and peripheral edema of the extremities. He was started on a loading dose of ASA (500 mg/day, administered in powder form), 32.5 g of γ-globulin, and 80 mg/day cyclosporine.

The patient’s fever subsided on the second day, leading to the discontinuation of γ-globulin. By the fourth day, the fever had completely resolved, and the ASA dosage was reduced to a maintenance dose of 80 mg/day, which was administered as mini-tablets. The patient began taking eight mini-tablets per day, administered as a single dose for 11 days and in divided doses for 14 days. The patient’s condition improved, allowing for the discontinuation of cyclosporine on the sixth day. The patient was discharged on the 9th day and advised to continue taking ASA mini-tablets for 60 days post-discharge.

Throughout treatment, no electrocardiographic abnormalities were observed, and cardiac function was adequately assessed using echocardiography. There was no evidence of coronary artery dilatation.

The patient was prescribed ASA mini-tablets for 65 days, but only 25 days (38.5% of the total prescription) were recorded in the medication diary. However, the medication diary confirmed that the patient was able to take the full amount of ASA mini-tablets during the recorded period. In addition, there was no mention of safety issues, such as swallowing difficulties or other adverse events, during this time.

### 3.5. Case 3

A 2-year-old girl presented with fever and ocular conjunctival hyperemia, along with an indeterminate rash and peripheral edema. There was no evidence of coronary artery disease. After excluding other potential diagnoses, KD was diagnosed based on four primary symptoms and a Gunma score of 6. The treatment regimen began with a loading dose of ASA (330 mg/day, administered as a powder formulation), 20 g of γ-globulin, and 40 mg/day cyclosporine.

The fever subsided on the second day, leading to the termination of γ-globulin treatment. Cyclosporine was discontinued on the sixth day after the fever was fully resolved and all major symptoms of KD abated. The ASA dosage was then reduced to a maintenance dose of 60 mg/day, administered as mini-tablets. The patient began taking six mini-tablets per day, administered as a single dose for the entire 62-day period. The patient responded well to treatment and was discharged on the eighth day of hospitalization. After discharge, the patient was instructed to continue taking ASA mini-tablets for 60 days.

During treatment, electrocardiography demonstrated transient ST-T changes but no other abnormal findings. Echocardiography revealed normal cardiac function, and there was no evidence of coronary artery dilation.

The patient had been prescribed ASA mini-tablets for a period of 63 days; however, only 62 days (98.4% of the total prescription) were recorded in the medication logbook. This is due to the unconfirmed status of the second medication record. Nevertheless, the medication diary confirmed that the patient took the entire quantity of ASA mini-tablets for all required days within the specified timeframe. No safety issues, such as swallowing difficulties, were observed.

## 4. Discussion

The form and dosage of pediatric medications vary according to the child’s age and developmental status [25,26]. Liquid formulations, such as syrups and suspensions, are the preferred option for younger children, whereas solid formulations such as tablets become more suitable as children age [3,27]. Recent evidence supports the use of mini-tablets as a pediatric dosage form [9,10,11,12,13,14,15]. However, the long-term efficacy of mini-tablets containing active pharmaceutical ingredients in pediatric disease management remains to be fully understood.

The most important finding of this study is that it demonstrated the feasibility of the repeated administration of ASA mini-tablets in the treatment of KD. Although the number of cases was limited to three, all the patients were able to take the prescribed doses without any problems, and no adverse events were reported. This result suggests that mini-tablets may be a useful option for pediatric patients whose medication adherence may be hindered by the taste of ASA.

The high degree of adherence and absence of adverse events observed in our study of three cases align with mounting evidence supporting mini-tablets as an effective pediatric dosage form. However, it is important to note that these findings are based on a very limited sample size and require confirmation through larger studies. Recent research has demonstrated that mini-tablets can effectively mask unpleasant tastes, a crucial factor in pediatric medication adherence [28,29]. This taste-masking ability, combined with ease of swallowing, highlights mini-tablets as a promising alternative to traditional powder formulations for drugs like acetylsalicylic acid.

Although the use of mini-tablets containing active ingredients has been studied in other countries, our research addresses a critical gap in pediatric formulation research specific to the Japanese population and healthcare context. The successful administration of ASA mini-tablets to children with KD corroborates findings from European studies showing the high acceptability of mini-tablets in young children [9,11].

The mini-tablets used in this study were prepared using ingredients that can be formulated in hospital pharmacies, although some excipients are specialized. This demonstrates the potential for preparing these mini-tablets in a hospital setting without the need for highly specialized equipment. The excipients used in this study are readily available to all medical institutions in Japan. In this study, although moisture-proofing coatings were not used in the formulation, the stability measures implemented were considered sufficient for the pilot study, given the short-term repetitive treatment and controlled storage conditions within the hospital.

This formulation utilized readily available hospital-grade ingredients, but manufacturing required specialized equipment for compression and quality control testing. Such equipment is not available in all hospitals, especially in developing countries. Therefore, the feasibility of mini-tablet production may vary depending on the resources available at each facility, and the widespread adoption of mini-tablets may require support for equipment acquisition and training of pharmacists with specialized knowledge and skills.

Furthermore, the type of beverage used during administration may significantly influence the drug’s stability, dissolution, and absorption. It is necessary to consider the effects of beverages on the stability and pharmacokinetics of mini-tablets. For example, acidic drinks may increase the solubility of ASA, potentially leading to faster absorption and higher peak plasma concentrations. Conversely, high-viscosity beverages like milk may delay disintegration time, possibly affecting drug absorption and bioavailability.

Future studies should evaluate the stability, dissolution, and pharmacokinetics of ASA mini-tablets when administered with various beverages and under different dosing regimens. This research will provide crucial guidance on optimal administration practices, ensuring that healthcare providers can offer clear instructions to patients and caregivers on how to take the medication effectively. These considerations are crucial for ensuring the efficacy and safety of ASA mini-tablets across various administration scenarios.

This study has several limitations that should be considered when interpreting the results. First, the sample size was very small, with only three patients included in the pilot study. This limited sample size restricts the statistical power of our findings and the generalizability of our conclusions to a broader population of pediatric patients with KD. A larger cohort is necessary to confirm the findings. Second, we focused on children aged 1 year and 11 months to 4 years. Subsequent research should expand the age range (6 months to 12 years) to assess long-term adherence, safety, and efficacy. Further research analyzing longer-term data and expanding the age range will allow for a more detailed evaluation of the long-term acceptability, safety, and adherence of mini-tablets. Third, a randomized controlled trial comparing ASA mini-tablets to conventional formulations (e.g., powder and syrup) is crucial to evaluate the tablets’ relative efficacy, acceptability, and adherence. Fourth, the medication adherence data in this study were derived from medication logbooks completed by caregivers, which may introduce recall bias and potential inaccuracies in reporting. Objective measures of medication adherence, such as electronic monitoring or drug level measurements, were not employed in this study. Furthermore, in this study, we were unable to conduct pharmacokinetic assessments, including the monitoring of ASA blood levels. Further studies should include pharmacokinetic evaluations to ensure bioequivalence between mini-tablets and conventional ASA formulations in pediatric patients with KD.

To date, Japan has not enacted regulations mandating the development of pediatric drugs or formulations. However, this criterion alone is insufficient for many pharmaceutical companies, particularly when the drug is low-cost [4,30]. The findings of this study suggest that mini-tablets have the potential to address the challenges associated with pediatric drug administration in Japan, where fine granules are frequently used [9]. The efficacy of mini-tablets in improving medication adherence and patient outcomes in pediatric populations warrants further exploration through larger studies [15]. This study represents the first clinical application of ASA mini-tablets in Japanese pediatric patients diagnosed with KD. By demonstrating the feasibility and safety of mini-tablet administration, this research lays the foundation for future studies aimed at improving pediatric healthcare and medication adherence.

## 5. Conclusions

This study demonstrated the successful administration of ASA mini-tablets for pediatric patients with KD. Our findings indicated high adherence rates and no safety issues associated with the use of mini-tablets in this population. The successful administration of ASA mini-tablets over the prescribed treatment period suggests that this formulation is a viable alternative to traditional liquid or powder formulations for pediatric patients. While further research with larger sample sizes is necessary to validate these findings and address the study limitations identified, this research provides valuable insights into the potential of mini-tablets to improve medication adherence and treatment outcomes in pediatric patients with KD. These results may have broader implications for pediatric drug development and could contribute to the advancement of age-appropriate formulations in pediatric pharmacotherapy. Mini-tablets have considerable potential to enhance medication adherence in children worldwide. It is anticipated that these will be developed not only in Japan but also through collaboration with global partners, including the European Pediatric Formulation Initiative.

## Figures and Tables

**Table 1:** Patient backgrounds and detailed clinical courses.

	Case 1	Case 2	Case 3
**Age at onset**	1 year and 11 months	4 years and 1 month	2 years and 3 months
**Sex**	Boy	Boy	Girl
**Height [cm]**	90.4	103.0	85.4
**Weight [kg]**	11.5	16.5	11.5
**Complications** *	None	None	None
**Medical history**	None	Hand, foot, and mouth disease (onset: unknown, cured)	None
**History of allergies**	None	None	None
**Concomitant medications**	None	None	None
**Kobayashi score [point]**	3	9	6
**ASA initial dose [mg/day]**	360 (36 MTs)	500 (powder)	330 (powder)
**Duration of initial treatment with ASA**	3 days	4 days	5 days
**ASA maintenance dose [mg/day]**	60 (6 MTs)	80 (8 MTs)	60 (6 MTs)
**Treatment duration with MT [days]**	68	65	63
**IVIG therapy [g/dose]**	25	32.5	20
**Cyclosporine therapy [mg/day]**	Not used	80	40
**Fever resolution [days]**	2	2	2
**Adherence rate [%]**	100	100	98.4
**Maximum continuous dosing**	37 **	25	62
**Adverse events**	None reported	None reported	None reported
**Coronary outcomes**	No dilation	No dilation	No dilation
**Beverages used**	Hojicha, jelly	Hojicha, water	Orange juice, hojicha, vegetable juice, water

MT: mini-tablet; IVIG: intravenous immunoglobulin. * indicates the presence of complications at the time of Kawasaki disease diagnosis. ** indicates that the patient forgot to take a scheduled dose on day 32. Thereafter, the timing of subsequent doses was adjusted by one day, and the regimen was continued until completion on day 69.

## Data Availability

The datasets used and/or analyzed during the study are available from the corresponding author upon reasonable request.

## Abbreviations

The following abbreviations are used in this manuscript:

ASA	Acetylsalicylic acid
KD	Kawasaki disease
MT	Mini-tablet
IVIG	Intravenous immunoglobulin

## References

[1] World Health Organization (2012). Development of Paediatric Medicines: Points to Consider in Formulation.

[2] Kojima J. (2015). Oral dosage forms for children: Acceptability and palatability. Yakugaku Zasshi.

[3] Saito J., Nakamura H., Walsh J., Yamatani A., Salunke S. (2022). A survey to understand parent/caregiver and children’s views on devices used for the administration of oral pediatric medicines in Japan. Children.

[4] Nakamura H., Ishikawa Y. (2014). How do Japanese children take their medicines, and what are pharmacists and paediatricians doing about it?. Int. J. Pharm..

[5] Lajoinie A., Henin E., Kassai B. (2015). Oral formulation of choice for children. Arch. Pediatr..

[6] Preis M. (2015). Orally disintegrating films and mini-tablets-Innovative dosage forms of choice for pediatric use. AAPS PharmSciTech.

[7] Almukainzi M., Araujo G.L.B., Löbenberg R. (2019). Orally disintegrating dosage forms. J. Pharm. Investig..

[8] Standing J.F., Tuleu C. (2005). Paediatric formulations—Getting to the heart of the problem. Int. J. Pharm..

[9] Mitsui N., Hida N., Kamiya T., Yamazaki T., Miyazaki K., Saito K., Saito J., Yamatani A., Ishikawa Y., Nakamura H. (2022). Swallowability of minitablets among children aged 6–23 months: An exploratory, randomized crossover study. Pharmaceutics.

[10] Miyazaki K., Hida N., Kamiya T., Yamazaki T., Murayama N., Kuroiwa M., Kurata N., Ishikawa Y., Yamashita S., Nakamura H. (2022). Comparative acceptability of mini-tablets, fine granules, and liquid formulations in young children: An exploratory randomized crossover study. J. Drug Deliv. Sci. Technol..

[11] Spomer N., Klingmann V., Stoltenberg I., Lerch C., Meissner T., Breitkreutz J. (2012). Acceptance of uncoated mini-tablets in young children: Results from a prospective exploratory cross-over study. Arch. Dis. Child..

[12] Klingmann V., Spomer N., Lerch C., Stoltenberg I., Frömke C., Bosse H.M., Breitkreutz J., Meissner T. (2013). Favorable acceptance of mini-tablets compared with syrup: A randomized controlled trial in infants and preschool children. J. Pediatr..

[13] Münch J., Kloft C., Farhan M., Fishman V., Leng S., Bosse H.M., Klingmann V. (2023). Acceptability, swallowability, palatability, and safety of multiple film-coated mini-tablets in children aged ≥2–<7 years: Results of an open-label randomised study. Pharmaceutics.

[14] Münch J., Meissner T., Mayatepek E., Wargenau M., Breitkreutz J., Bosse H.M., Klingmann V. (2021). Acceptability of small-sized oblong tablets in comparison to syrup and mini-tablets in infants and toddlers: A randomized controlled trial. Eur. J. Pharm. Biopharm..

[15] Zuccari G., Alfei S., Marimpietri D., Iurilli V., Barabino P., Marchitto L. (2022). Mini-tablets: A valid strategy to combine efficacy and safety in pediatrics. Pharmaceuticals.

[16] Thomson S.A., Tuleu C., Wong I.C.K., Keady S., Pitt K.G., Sutcliffe A.G. (2009). Minitablets: New modality to deliver medicines to preschool-aged children. Pediatrics.

[17] PubChem Compound Summary Aspirin. https://pubchem.ncbi.nlm.nih.gov/compound/Aspirin.

[18] Singh S., Vignesh P., Burgner D. (2015). The epidemiology of Kawasaki disease: A global update. Arch. Dis. Child..

[19] Miura M., Ayusawa M., Fukazawa R., Hamada H., Ikeda S., Ito S., Kanai T., Kobayashi T., Suzuki H., Yamamura K. (2021). Guidelines for medical treatment of acute Kawasaki disease (2020 revised version). J. Pediatr. Cardiol. Card. Surg..

[20] Burns J.C., Glodé M.P. (2004). Kawasaki syndrome. Lancet.

[21] Newburger J.W., Takahashi M., Burns J.C. (2016). Kawasaki disease. J. Am. Coll. Cardiol..

[22] McCrindle B.W., Rowley A.H., Newburger J.W., Burns J.C., Bolger A.F., Gewitz M., Baker A.L., Jackson M.A., Takahashi M., Shah P.B. (2019). Correction to: Diagnosis, Treatment, and Long-Term Management of Kawasaki Disease: A Scientific Statement for Health Professionals from the American Heart Association. Circulation.

[23] Nakamura Y., Yashiro M., Uehara R., Sadakane A., Chihara I., Aoyama Y., Kotani K., Yanagawa H. (2010). Epidemiologic features of Kawasaki disease in Japan: Results of the 2007–2008 nationwide survey. J. Epidemiol..

[24] Hida N., Yamazaki T., Fujita Y., Noda H., Sambe T., Ryu K., Mizukami T., Takenoshita S., Uchida N., Nakamura A. (2023). A Study on Pharmacokinetics of Acetylsalicylic Acid Mini-Tablets in Healthy Adult Males-Comparison with the Powder Formulation. Pharmaceutics.

[25] Bartelink I.H., Rademaker C.M.A., Schobben A.F.A.M., van den Anker J.N. (2006). Guidelines on paediatric dosing on the basis of developmental physiology and pharmacokinetic considerations. Clin. Pharmacokinet..

[26] Ivanovska V., Rademaker C.M.A., van Dijk L., Mantel-Teeuwisse A.K. (2014). Pediatric drug formulations: A review of challenges and progress. Pediatrics.

[27] Alessandrini E., Brako F., Scarpa M., Lupo M., Bonifazi D., Pignataro V., Cavallo M., Cullufe O., Enache C., Nafria B. (2021). Children’s preferences for oral dosage forms and their involvement in formulation research via EPTRI (European paediatric translational research infrastructure). Pharmaceutics.

[28] Golhen K., Buettcher M., Kost J., Huwyler J., Pfister M. (2023). Meeting challenges of pediatric drug delivery: The potential of orally fast disintegrating tablets for infants and children. Pharmaceutics.

[29] Sanjay L.R., Ashokbhai M.K., Ghatole S., Roy S., Kashinath K.P., Kaity S. (2025). Strategies for beating the bitter taste of pharmaceutical formulations towards better therapeutic outcomes. RSC Pharm..

[30] Saito J., Akabane M., Nakamura H., Ishikawa Y., Yamatani A. (2019). Oral drug compounding in pediatric patients: A Japanese perspective. J. Pharmacol. Pharm. Res..

